# No evidence for viral small RNA production and antiviral function of Argonaute 2 in human cells

**DOI:** 10.1038/s41598-019-50287-w

**Published:** 2019-09-24

**Authors:** Susan Schuster, Gijs J. Overheul, Lisa Bauer, Frank J. M. van Kuppeveld, Ronald P. van Rij

**Affiliations:** 1 Department of Medical Microbiology, Radboud University Medical Center, Radboud Institute for Molecular Life Sciences, Nijmegen, The Netherlands; 2 Department of Infectious Diseases & Immunology, Faculty of Veterinary Medicine, Utrecht University, Utrecht, The Netherlands

**Keywords:** Innate immunity, Virus-host interactions, RNAi

## Abstract

RNA interference (RNAi) has strong antiviral activity in a range of animal phyla, but the extent to which RNAi controls virus infection in chordates, and specifically mammals remains incompletely understood. Here we analyze the antiviral activity of RNAi against a number of positive-sense RNA viruses using Argonaute-2 deficient human cells. In line with absence of virus-derived siRNAs, Sindbis virus, yellow fever virus, and encephalomyocarditis virus replicated with similar kinetics in wildtype cells and Argonaute-2 deficient cells. Coxsackievirus B3 (CVB3) carrying mutations in the viral 3A protein, previously proposed to be a virus-encoded suppressor of RNAi in another picornavirus, human enterovirus 71, had a strong replication defect in wildtype cells. However, this defect was not rescued in Argonaute-2 deficient cells, arguing against a role of CVB3 3A as an RNAi suppressor. In agreement, neither infection with wildtype nor 3A mutant CVB3 resulted in small RNA production with the hallmarks of canonical vsiRNAs. Together, our results argue against strong antiviral activity of RNAi under these experimental conditions, but do not exclude that antiviral RNAi may be functional under other cellular, experimental, or physiological conditions in mammals.

## Introduction

The RNA interference (RNAi) pathway mediates antiviral immunity in insects and plants, while the type I interferon (IFN) system serves as the first line of defense in mammals. Antiviral RNAi in insects is characterized by the recognition and processing of viral double-stranded RNA (dsRNA) into virus-derived small interfering RNAs (vsiRNAs) of 21-nt in length by the RNase III enzyme Dicer-2 and its co-factor R2D2^1,2^. Viral siRNAs are loaded onto an Argonaute-2 containing RNA-induced silencing complex (RISC) that mediates the degradation of one strand (passenger) and cleavage of complementary viral target RNAs using the retained strand as a guide^1,2^. The RNAi pathway is evolutionary conserved, with variations, in eukaryotes^3^. Therefore, the machinery to process dsRNA into small silencing RNAs is also present in mammals, but primarily seems to function in the microRNA (miRNA) pathway^4^. In *Drosophila*, miRNA and siRNA biogenesis and function depend on distinct Dicer and Argonaute proteins^5,6^, while mammals only encode a single Dicer protein that mediates both miRNA and siRNA biogenesis^2,7^. Moreover, all four members of the Argonaute (AGO) family in mammals engage in microRNA-guided gene silencing, whereas only AGO2 mediates siRNA-dependent target RNA cleavage^8–14^.

Differentiated mammalian cells encode pattern-recognition receptors that activate the type I IFN response upon detection of viral pathogen-associated molecular patterns. For example, the DEAD box helicases RIG-I (retinoic acid-induced gene I) and MDA5 (melanoma differentiation-associated protein 5) recognize viral dsRNA and other non-self RNA signatures and activate a signaling cascade that leads to the activation of type I IFN genes^15^. Type I IFNs then bind the interferon-α/β receptor to activate the JAK/STAT pathway in a paracrine and autocrine fashion, resulting in the production of hundreds of interferon stimulated genes (ISGs)^16,17^. These ISGs act broadly antiviral, for example by inhibiting transcription (MX1, TRIM5), translation (PKR, IFIT family, OASL), replication (IFIT family, OAS1/2/3)^18,19^, or by degrading RNA and inducing apoptosis (RNaseL)^18,19^.

Although RNAi is evolutionary conserved, there is an ongoing debate whether and to what extent RNAi contributes to antiviral defense in mammalian hosts^20–28^. While virus infection of somatic cells resulted in little accumulation of virus-derived siRNAs in several studies^29–32^, others have found evidence for antiviral activity of RNAi under certain experimental conditions^22,33^. Indeed, a growing body of evidence appears to set the stage and, at the same time, limitations for a detectable RNAi response in mammals. First, an effective IFN response could mask or inhibit RNAi in differentiated cells. A recent study has shown that interferon-deficient differentiated cells are able to process dsRNA and silence a reporter construct^23^, although in another study, deletion of the cytoplasmic dsRNA sensors RIG-I and MDA5 was not sufficient to uncover antiviral RNAi^30^. Moreover, the RIG-I-like receptor LGP2 interacts with Dicer to inhibit processing of long dsRNA into siRNAs, indicating an intricate interplay between RNAi and the IFN response^34^. Second, vsiRNAs accumulated to detectable levels in mouse embryonic stem cells (mESCs) infected with encephalomyocarditis virus (EMCV), whereas no vsiRNAs could be detected after differentiation into an ectodermal cell lineage^22^. Embryonic stem cells are unable to produce type I IFNs and induce low, if any, ISG expression upon virus infection or stimulation with dsRNA mimics, suggesting that the interferon response is incompatible with pluripotency^35–38^. The inability of stem cells to mount a productive interferon-based antiviral response could therefore contribute to a functional RNAi-mediated antiviral response. Third, vsiRNAs of ~22 nt, the characteristic length of mammalian Dicer products, could be detected when using viruses lacking their viral suppressor of RNAi (VSR) in several experimental systems including somatic cells, embyonic stem cells, and suckling mice^21,33,39^. In contrast, no virus-derived siRNAs were detected when using wildtype viruses, suggesting that VSRs potently block siRNA production. This has now been observed in the context of three RNA viruses and their VSRs: Nodamura virus (*Nodaviridae*) lacking B2, influenza A virus (*Orthomyxoviridae*) lacking NS1, and human enterovirus 71 (HEV71) (*Picornaviridae*) defective in 3A function^21,33,39^.

In this study, we analyze antiviral activity of RNAi in differentiated cells in response to wildtype RNA viruses and a virus defective in its proposed VSR using Argonaute 2 deficient cell lines. We used wild-type viruses from three distinct virus families, Sindbis virus (SINV, *Togaviridae*), yellow fever virus 17D vaccine strain (YFV, *Flaviviridae*), and encephalomyocarditis virus (EMCV, *Picornaviridae)*. In addition, we analyzed Coxsackievirus B3 (CVB3, *Picornaviridae*) carrying mutations (D24A K41Q) analogous to the ones that render HEV71 3A RNAi suppression deficient. In this experimental set-up, we fail to detect strong antiviral activity of RNAi.

## Results and Discussion

### Characterization of AGO2 knockout HeLa cells

**Figure 1:**
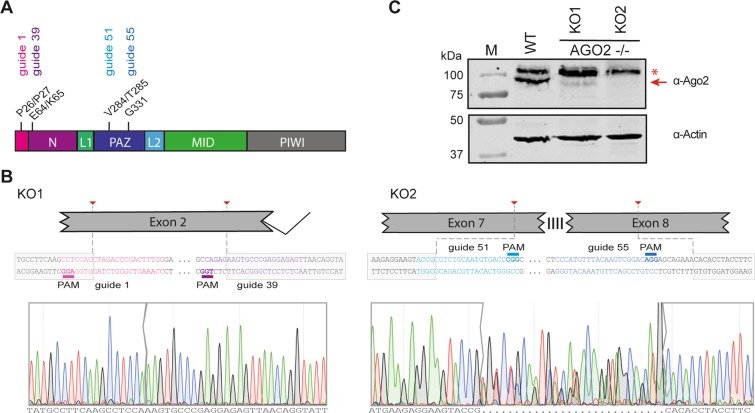
Generation of AGO2 knockout cell lines using CRISPR/Cas9 technology. (**A**) Schematic representation of the AGO2 gene, (**B**) location and sequences of guide RNAs, and the sequence of the targeted region of two AGO2 knockout HeLa cell clones (KO1 and KO2). (**C**) Western blot analysis of AGO2 (top panel) and Actin (lower panel) in wildtype control (WT) cells and AGO2 KO clones. The red arrow indicates AGO2, asterisk indicates a non-specific product. M, size marker with molecular mass (in kDa) indicated. The full-length blot is presented in Supplementary Fig. S5.

We used CRISPR/Cas9 technology using two guide RNA combinations to engineer AGO2 deficient HeLa R19 cells and selected two clones for further analyses. One guide RNA combination (guide RNAs 1 and 39) introduced a 114-bp in-frame deletion into exon 2 (Fig. 1A,B) in 5 out of 48 single-cell clones, one of which was used for further study (referred to as KO1). A second AGO2 mutant cell clone, referred to as KO2, was generated by an independent combination of two guide RNAs (guide 51 and guide 55). This guide combination yielded 32 single-cell colonies, of which only one (KO2) carried deletions around the predicted cleavage sites in exon 7 and exon 8 (Fig. 1B). AGO2 was undetectable by western blot analysis in KO2, whereas AGO2 expression was strongly reduced in KO1 (Fig. 1C, red arrow). Putative residual AGO2 expression detectable in KO1 (Fig. 1C) was not consistently detected (Supplementary Fig. S5A).

**Figure 2:**
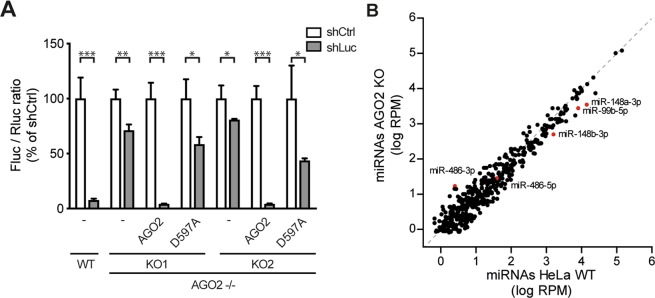
Functional validation of AGO2 knockout cells. (**A**) RNAi reporter assay in HeLa WT control and two AGO2 KO cell clones. Cells were transfected with expression plasmids encoding firefly luciferase (Fluc), Renilla luciferase (Rluc), either Fluc specific shRNA (shLuc) or a non-targeting control shRNA (shCtrl), along with expression plasmids encoding wildtype AGO2 or slicer deficient AGO2 D597A where indicated. Fluc counts were normalized to Rluc counts and expressed as a percentage of the non-targeting control shRNA. Data are presented as means and SD of three biological replicates. Data are from one experiment representative of three experiments. *P < 0.05, **P < 0.01, ***P < 0.001, Student’s t-test. (**B**) Scatter plot representing miRNA levels in WT and AGO2 KO2 HeLa cells. Reads are normalized to library size. Averaged data from EMCV infected HeLa cells at 8 and 10 hpi are presented (plots for the individual time points in Supplementary Fig. 2). Selected miRNAs are indicated in red.

To functionally validate the AGO2 deficient cell lines, we used an established RNAi reporter assay, in which expression of firefly luciferase (Fluc) was silenced by a short hairpin RNA (shRNA), using non-targeting shRNA as a control and Renilla luciferase to normalize the signal^30^. In wildtype control (WT) cells, Fluc expression was efficiently silenced by the sequence specific shRNA (13-fold relative to non-targeting shRNA), whereas silencing was virtually abolished in AGO2 KO1 and KO2 cells, respectively (Fig. 2A). To validate that RNAi deficiency was due to the absence of AGO2, both AGO2 KO cell lines were transfected with an expression plasmid encoding wildtype AGO2 or a catalytic slicer deficient mutant (D597A)^11^. As expected, expression of wildtype AGO2, but not slicer deficient AGO2, efficiently rescued the defect in RNAi in both AGO2 KO lines (Fig. 2A). We speculate that the minor, ~2 fold silencing by slicer deficient AGO2 is caused by a miRNA-like mechanism, as the D597A mutation affects the catalytic core responsible for target RNA cleavage^40^. These results indicate that the generated clonal AGO2 KO cell lines lack a functional siRNA-mediated RNAi response.

### No major deregulation of cellular miRNAs in AGO2 knockout cells

We previously reported absence of vsiRNA production in HeLa cells upon infection with yellow fever virus (YFV), Coxsackie virus B3 (CVB3) and Sindbis virus (SINV), both in wildtype cells and cells lacking the RNA helicases RIG-I and MDA5^30^. To extend these observations, we analyzed small RNAs in WT control and AGO2 KO HeLa cells upon infection with encephalomyocarditis virus (EMCV, strain mengovirus), which has previously been shown to produce vsiRNAs in mESCs^22^. Processing of viral dsRNA into vsiRNAs would give rise to viral small RNA profiles that are enriched for 22-nt sequence reads, derived from both the viral positive (+) and negative (−) sense RNA in approximately equal numbers. Moreover, EMCV-derived vsiRNAs in mESCs predominantly derive from the 5′end of the viral genome with evidence for phasing with ~22-nt periodicity, indicative of sequential Dicer cleavages from the ends of the genome^22^. In contrast, we found that EMCV-derived viral small RNA are not enriched for 22-nt reads and predominantly derive from the positive (+) viral RNA strand, suggesting that these are viral breakdown products (Supplementary Fig. S1), similar to our previous observations with YFV, CVB3, and SINV^30^. These results indicate that in this cellular context EMCV dsRNA is not efficiently targeted by Dicer to produce vsiRNAs.

AGO2 is one of four AGO genes in humans, which seem to act redundantly in the miRNA pathway. We thus analyzed cellular miRNAs in WT and AGO2 KO cells (at 8 and 10 h after EMCV infection) and observed a strong correlation of the majority of miRNAs, although many miRNAs accumulate to slightly lower levels in AGO2 KO cells (*R*^2^ = 0.926, y = 0.9629 × −0.0592, linear regression, Fig. 2B, Supplementary Fig. S2), as has been observed before^41–43^. Notably, AGO2 KO HeLa cells recapitulate previous observations of specific AGO2-dependent miRNAs, such as miR-148a-3p and miR-148b-3p that have been shown to be reduced in AGO2 deficient liver cells^44^. Moreover, miR-99b-5p was reported to be enriched in AGO2 over other AGO proteins^13,14^ and it is possible that such miRNAs are destabilized under AGO2 KO conditions, as observed in our data. Biogenesis of two miRNAs depend on the catalytic activity of AGO2, miR-451 and miR-486^41,45–47^. In case of miR-451, AGO2 cleavage is required for pre-miRNA maturation independently from Dicer, whereas for miR-486, AGO2 cleavage removes the 3p passenger strand. As a consequence, miR-486-3p accumulates to higher levels in the absence of AGO2, without dramatically affecting miR-486-5p. Although miR-451 was only lowly expressed in our dataset (in line with its tissue specific expression in erythroid cells), we observed similar effects of AGO2 KO on miR-486 in our data. Together, these observations validate that our AGO2 KO cells are RNAi deficient.

### RNA viruses replicate with similar kinetics in control and AGO2 knockout cells

**Figure 3:**
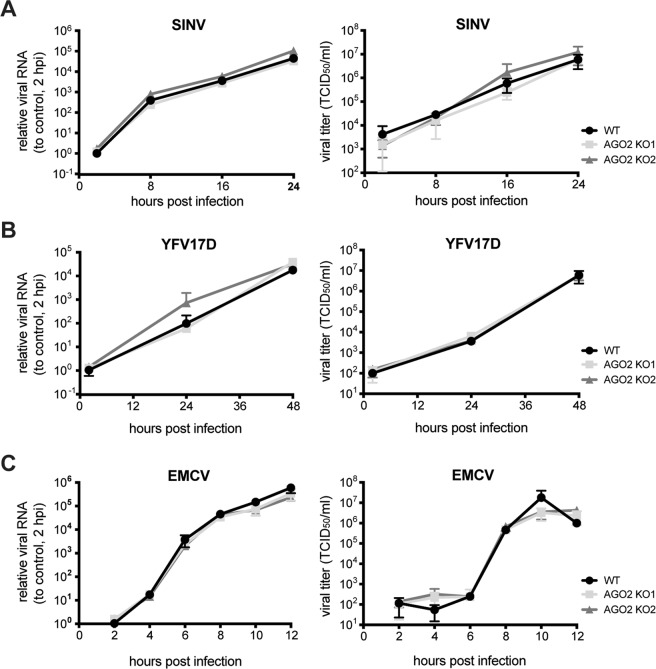
Viral replication kinetics in wildtype and AGO2 knockout cells. Intracellular viral RNA levels (left panels) and infectious viral titers (left panels) in the supernatant of WT (black) and AGO2 KO HeLa cells (light and dark grey) infected with (**A**) Sindbis virus (SINV, MOI 0.01), (**B**) yellow fever virus 17D (YFV17D, MOI 0.1), and (**C**) encephalomyocarditis virus (EMCV, MOI 1). Viral RNA was quantified by RT-qPCR, normalized to the house-keeping gene actin and expressed relative to viral RNA levels at 2 hpi. Data are from one experiment representative of three experiments, each performed with n = 3 biological replicates, presented as means and SD. hpi, hours post infection.

Although our results indicate that viral dsRNA is not efficiently processed into vsiRNAs^30^, we cannot exclude the possibility that vsiRNAs are below the detection level in a cellular system with abundant viral breakdown products. We therefore analyzed viral replication kinetics of a panel of viruses in WT and AGO2 KO cells, under the hypothesis that an antiviral effector function of AGO2 would be evident from an increase in viral accumulation in the absence of AGO2. We selected a panel of RNA viruses from different virus families (SINV, YFV, EMCV) that we previously analyzed for vsiRNA production^30^ (Supplementary Fig. S1) and assessed internal viral RNA replication using RT-qPCR and quantified infectious viral particles in the supernatant by endpoint serial dilution assay. For all viruses, we observed similar intracellular replication kinetics and virus accumulation in the supernatant of WT and AGO2 KO cells (Fig. 3). Under some conditions, slightly enhanced virus production was observed in AGO2 KO cells (e.g. for SINV in AGO2 KO2 cells at 24 hours post infection, hpi), which was not consistent between the two individual AGO2 KO lines (Fig. 3A) or between independent experiments (data not shown). These results indicate that AGO2 is not involved in a robust antiviral response in these cells.

Recent studies found that processing of (viral) dsRNA into siRNAs is inhibited or masked by the IFN response^23,34^. Moreover, innate antiviral pathways regulate AGO2 function and, conversely, ISG expression is under control of miRNAs^48^. To assess whether the type I IFN response is altered in the absence of AGO2 in our experimental system, we analyzed IFNβ and ISG15 mRNA expression after stimulation of WT and AGO2 KO cells with poly (I:C) or the panel of RNA viruses. AGO2 deficient KO2 cells stimulated with poly (I:C) show a small increase in IFNβ and ISG15 mRNA expression compared to WT cells, whereas no significant differences were observed for KO1 cells (Supplementary Fig. S3). For SINV infection, expression of IFNβ and ISG15 was higher in KO2 cells compared to WT cells at all time points, while in KO1 cells increased ISG15 expression was only observed at later time points (16 and 24 hpi) (Supplementary Fig. S3). Likewise, during YFV infection, increased expression of IFNβ (48 h) and ISG15 (24 h and 48 h) was observed in AGO2 KO2 cells, but not KO1 cells. The leader protein of EMCV is a strong suppressor of the interferon response^49^. In agreement, we find only mild induction of IFNβ and ISG15 expression upon EMCV infection, and these responses did not significantly differ between WT and AGO2 KO cells. Together these observations indicate an increased IFN response in AGO2 KO2 cells, consistent with previous observations^48^. It is currently unclear whether the discrepancy between AGO2 KO1 and KO2 cells is due to clonal effects or to the relatively small in-frame deletion and putative residual AGO2 expression in KO1 cells.

### No evidence for an RNAi suppressive activity of CVB3 3A protein

The 3A protein of human enterovirus 71 (HEV71) was recently proposed to bind dsRNA and inhibit RNAi by suppressing Dicer-dependent siRNA production^39^. To confirm and extend these observations, we analyzed RNAi suppression by the 3A proteins of HEV71, CVB3, and poliovirus type 1, all of which belong to *Enterovirus* genus within the *Picornaviridae* family. RNAi suppression was analyzed after transfection of expression plasmids encoding 3A, followed by transfection of plasmids encoding luciferase and shRNAs as described earlier (Fig. 1C). RNAi-mediated silencing was as efficient in cells expressing 3A as in cells transfected with control plasmids, whereas RNAi was suppressed in cells expressing a known VSR, Nodamura virus B2 protein^50^, as expected (Supplementary Fig. S4A). This was not due to inefficient expression of the 3A proteins in HeLa cells, as verified by western blot (Supplementary Fig. S4B). These observations suggest that 3A does not act as an RNAi suppressor under these conditions, although we cannot rule out that our transfection-based assay could miss subtle VSR activity.

**Figure 4:**
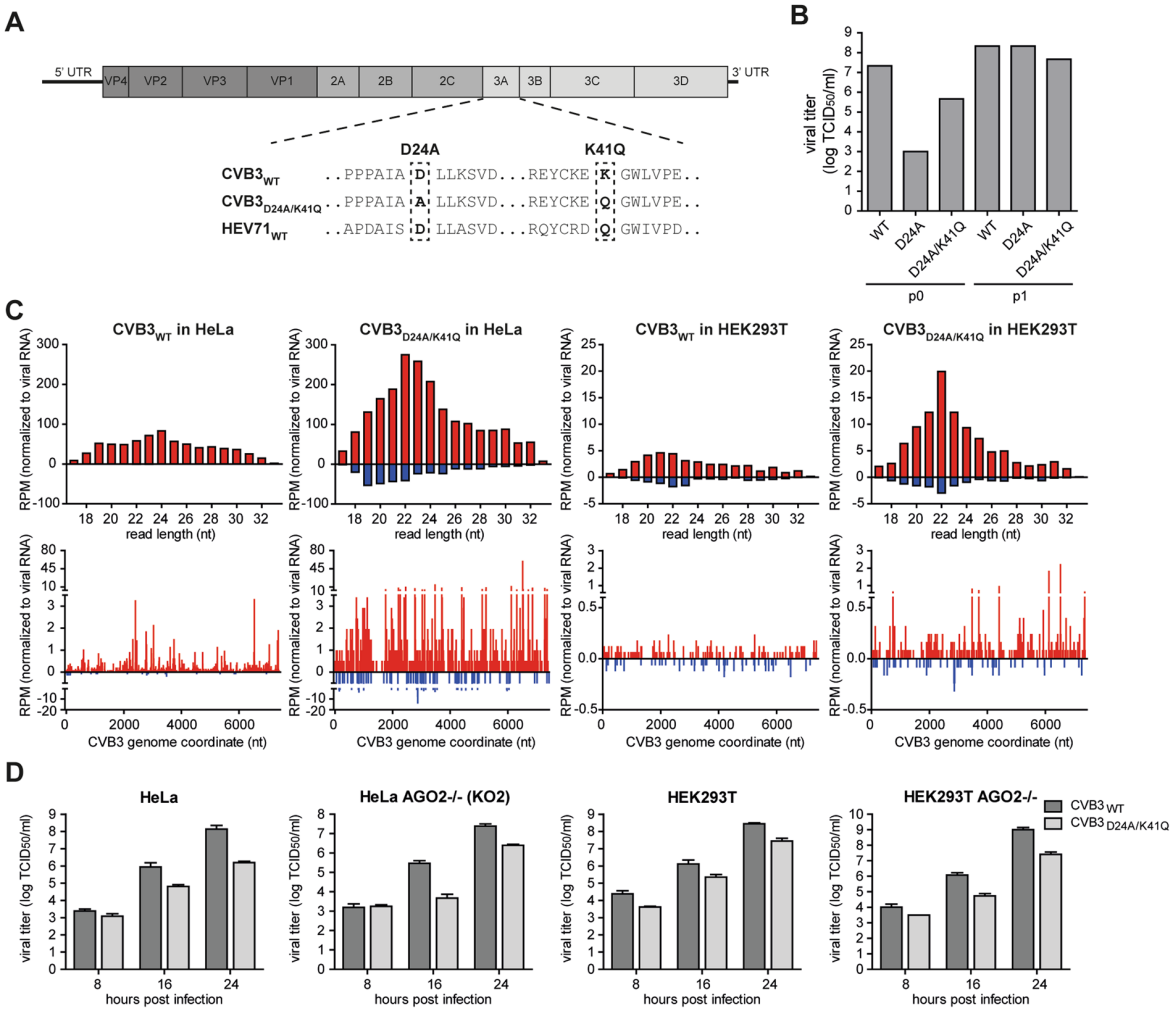
No evidence for an RNAi suppressive function of CVB3 3A protein. (**A**) Schematic representation of the CVB3 genome with an alignment of the 3A sequence with the HEV71 3A sequence, highlighting mutations introduced into CVB3 3A. (**B**) Viral titers of passage 0 (p0) and passage 1 (p1) CVB3 virus stocks harvested from HeLa cells. Viral titers were determined by end-point serial dilution on HeLa cells. (**C**) Top panels, size profiles of small RNAs mapping to the positive viral RNA strand (red) or negative RNA strand (blue) of CVB3. Reads were mapped allowing one mismatch and normalized to the total library size and to viral RNA levels in the sample used to prepare the sequencing libraries (relative to CVB3_WT_ infection in each cell line). Lower panels, distribution of 21–23 nt viral small RNAs across the CVB3 genome. 5′ positions of normalized reads are plotted. (**D**) Viral titers of supernatant harvested from CVB3_WT_ and CVB3_D24A/K41Q_ infected WT or AGO2 KO HeLa and HEK293T cells at 8, 16 and 24 hpi with p0 virus. Bars represent means and SD of 3 biological replicates.

Qiu *et al*. identified a point mutation (D23A) that rendered the HEV71 3A protein RNAi suppression defective^39^. Engineering this mutation into the genome of HEV71 introduced a strong replication defect that was rescued, albeit to a small extent, in a Dicer deficient background, providing genetic support for the importance of 3A as a VSR^39^. To confirm these observations, we set out to generate a HEV71 mutant carrying the D23A mutation. This mutation was inserted into an infectious cDNA clone of HEV71 strain BrCr, but unfortunately no virus was obtained following transfection of cells with run-off RNA transcripts. As enterovirus 3A is a conserved protein, we then aimed to analyze replication and small RNA profiles of CVB3 with an analogous mutation (D24A in CVB3). In CVB3, D24 forms an intermolecular salt bridge with K41 in the 3A dimer^51^. Previous work indicated that the D24A mutation renders CVB3 non-infectious and required a second-site mutation to restore infectivity^51^. One such second site mutations is Q41A, which is the natural amino acid in HEV71A. We therefore introduced either D24A, alone or in combination with K41Q into CVB3 (Fig. 4A). Transfection of *in vitro* transcribed RNA of CVB3_D24A_ and CVB3_D24A/K41Q_ produced respectively >4-log and >1-log less infectious virus than WT CVB3 RNA (passage 0), indicating that these mutants had severe replication defects and confirming that the second-site K41Q mutation restores infectivity. After a single passage in HeLa cells, however, viral titers were undistinguishable from WT virus titers (Fig. 4B), and Sanger sequencing indicated that 100% of the passaged virus reverted to the wildtype D24 residue for CVB3_D24A_, whereas passage 1 of the CVB3_D24A/K41Q_ mutant contained a mix of wildtype and mutant sequences (data not shown). We therefore used the passage 0 virus stock and verified the presence of the mutation by Sanger sequencing in all subsequent experiments.

We next analyzed viral small RNA profiles of HeLa and HEK293T cells infected with CVB3_WT_ and CVB3_D24A/K41Q_. Should 3A protect viral dsRNA from Dicer-mediated processing, it is expected that canonical vsiRNAs can be detected during CVB3_D24A/K41Q_ infections. For CVB3_WT_ in HeLa cells, we observed a broad distribution of viral small RNAs with a slight enrichment in the size range of 22–25 nt on the positive (+) viral RNA strand, but almost no negative (−) strand-derived reads (sense/antisense ratio of 99.0 for 21–23 nt reads, Fig. 4C). Likewise, in CVB3_WT_ infected HEK293T cells, viral small RNAs show a broad size distribution, slightly enriched in the size range of 20–22 nt, but the reads predominantly derived from the (+) RNA strand (sense/antisense ratio of 2.7 for 21–23 nt reads). Strikingly, in CVB3_D24A/K41Q_ infections, (+) strand reads in HeLa and both (+) and (−) strand reads in HEK293T cells were enriched for 22-nt reads. Yet, mutant virus-derived small RNAs remained biased towards the (+) strand in both cell types (sense/antisense ratio of 6.6 and 7.0 for 21–23 nt reads in HeLa and HEK293T, respectively), although this bias was less pronounced than in CVB3_WT_ infected HeLa cells. In all cases, 21–23 nt small RNAs covered the entire viral genome without prominent enrichment at the 5′ or 3′ termini or evidence for phasing (Fig. 4C), unlike previous observations for HEV71_D23A_^39^.

Given the difference in replication of wildtype and mutant CVB3, we normalized viral small RNA counts to relative viral RNA levels. Notably, normalized viral small RNA counts were higher across the entire size range (17–33 nt) in CVB3_D24A/K41Q_ infections than in CVB3_WT_ infections, both in HeLa and HEK293T cells (3.4 and 2.5-fold, respectively). Together these results suggest that the 3A mutation renders viral RNA more accessible to cellular nucleases, which in HEK293T cells results in the production of small RNAs in the size range of Dicer products in mammals. Yet, the strong (+) strand bias argues against Dicer-dependent biogenesis of these small RNAs.

To analyze whether these small RNAs contribute to antiviral defense and whether the replication defect of CVB3_D24A/K41Q_ is due to defective VSR activity, we performed a genetic rescue experiment. To this end, we compared replication of CVB3_D24A/K41Q_ with CVB3_WT_ in WT and AGO2 KO cells, both in a HeLa and HEK293T cell background^52^. If RNAi restricts replication of the CVB3_D24A/K41Q_, it is expected that the replication defect is reduced or even absent under conditions in which AGO2 is inactivated. We observed that CVB3_D24A/K41Q_ indeed replicated to lower viral titers compared to CVB3_WT_, but this defect was not rescued in AGO2 deficient cells (Fig. 4D). Specifically, the replication defect of CVB3_D24A/K41Q_ was similar in WT and AGO2 deficient cells across all time points, both in the HeLa and HEK293T backgrounds (Fig. 4D).

Together, these results are not consistent with a function of CVB3 3A in suppressing RNAi. The membrane-associated enterovirus 3A protein is essential for remodeling of secretory pathway membranes into replication organelles and the D24 residue is essential for 3A dimerization, membrane remodeling, and viral RNA replication^53^, as also evident in the replication defect of CVB3_D24A_ and reversion to wildtype sequence in our study. The previously reported detection of viral (−) RNA reads in HEV71_D23A_ infection^39^, and the increased levels of viral small RNA in CVB3_D24A/K41Q_ infection in our study (Fig. 4C) may be due to less efficient production of replication organelles and increased exposure of replicating viral RNA to cellular nucleases.

## Conclusion

The antiviral potential of RNAi remains incompletely understood. In this study, we fail to obtain evidence for a strong antiviral function of RNAi using AGO2 deficient HeLa cells, in line with failure to detect vsiRNAs for the viruses studied in this cellular context (Supplementary Fig. S1)^30^. These observations match those of Bogerd *et al*.^29^, who did not find enhanced replication of a broad panel of RNA viruses and a DNA virus in Dicer deficient 293T cells, and with those from Maillard *et al*.^23^, who did not observe consistent increases in replication of Semliki forest virus, influenza A virus and EMCV in slicer defective AGO2 fibroblasts compared to cells with slicer competent AGO2. Yet, others have obtained evidence that RNAi may control viral infections under certain cellular conditions^21,22,33,39^ and we cannot exclude that the choice of cells in our study has affected our results.

The absence of a viral suppressor of RNAi was found to render viruses sensitive to antiviral RNAi^21,33,39^, which we have tried to extend here by analyzing replication of a CVB3 variant carrying mutations in 3A, previously proposed to be a VSR in HEV71. Unfortunately, our data do not support a role of CVB3 3A as an RNAi suppressor. Thus, the precise cellular conditions required for antiviral RNAi in mammals remain to be defined.

## Materials and Methods

### Cell culture and viruses

HeLa R19 cells were cultured in Dulbecco’s modified Eagle medium (DMEM) with 4.5 g/L D-glucose (Gibco 41965-039), 1% Pen Strep (Gibco 15070-063) and 10% fetal bovine serum (Gibco 10500-064) in 5% CO_2_ at 37 °C. For poly (I:C) transfections, cells were seeded at a density of 7.5 × 10^4^ cells per well in 500 µL medium in a 24-well plate. The next day, the cells were transfected with 200 ng or 2 μg of poly (I:C) (GE Healthcare) using FuGENE 6 (Promega E2693). RNA was isolated after an incubation for 6 h. HEK293T and BHK-21 cells were cultured in Dulbecco’s modified Eagle medium (DMEM) with 4.5 g/L D-glucose (Gibco 41965-039), 1% Pen Strep (Gibco 15070-063) and 10% fetal bovine serum (Gibco 10500-064) in 5% CO_2_ at 37 °C. RD cells (kindly provided by Johan Neyts, University of Leuven) were cultured in DMEM (Lonza) supplemented with 10% fetal bovine serum (Lonza).

Sindbis virus was derived from the pTE-3′2J infectious cDNA^54^, produced by transfection of *in vitro* transcribed RNA into BHK-21 cells and titered on HeLa cells, as previously described^55^. EMCV (strain mengovirus) was produced by transfection of *in vitro* transcribed RNA generated from cDNA clone pM16.1 into BHK-21 cells and titered on BHK-21 cells, as previously described^56^. YFV 17D was kindly provided by Ella Driessen (dept. Medical Microbiology, Radboud University Medical Center) and produced and titered in BHK-21 cells. Briefly, BHK-21 cells were inoculated with YFV 17D at an MOI 0.2 in a T75 flask, and supernatant was harvested at 72 and 96 hpi, centrifuged at 300 x *g*, and stored in aliquots at −80 °C. To analyze replication kinetics, WT and AGO2 KO cells were plated in full DMEM medium overnight, inoculated with virus in fresh medium for 2 h, after which the medium was replaced with fresh medium. Supernatant and cells were harvested at the indicated time points for titration and RT-qPCR.

The D23A mutation in the viral protein 3A was introduced into the infectious clone pEV71-(BrCr-Tr)^57^ using site-directed mutagenesis with the following oligonucleotides: 5′-TGCCATTAGTGCTTTGCTCGCTAG-3′ and 5′-TCTGGGGCTGGCTTTTCT-3′. The presence of the mutation in the infectious clone was verified with Sanger sequencing. Next, a 1079 bp fragment containing the desired mutation was isolated using the enzymes *Sal*I and *Spe*I and reintroduced into the original non-mutagenized pEV71-(BrCr-Tr) plasmid. The plasmid was linearized with *Mlu*I and *in vitro* RNA transcribed. *In vitro* transcribed RNA was transfected into Hela R19 and RD cells to obtain virus, but no virus could be obtained after passaging lysates 3 times on either HelaR19 or RD cells.

The CVB3 infectious clone p53CB3/T7^51^ contains the full-length sequence of CVB3 (Nancy strain) driven by the T7 polymerase promoter. The D24A and K41Q mutations were introduced into 3A by site-directed mutagenesis using In-Fusion HD Cloning Plus (Takara 638910) with primers 5′-GGCCCTGCTCAAATCGGTAG-3′ and 5′-GATTTGAGCAGGGCCGCAATG-3′ for 3A D24A and 5′-GAACAAGGATGGTTGGTTCC-3′ and 5′-CAACCATCCTTGTTCTTTGCAG-3′ for 3A K41Q. The plasmids were linearized with *Mlu*I and RNA was transcribed using the RiboMAX Large Scale RNA Production System (Promega P1300). To produce virus, HeLa cells were seeded at a density of 1 × 10^6^ cells per well in a 6-well plate and transfected with 2.5 µg of CVB3 *in vitro* transcribed RNA using Effectene Transfection Reagent (Qiagen 301427). Culture medium was harvested at 24 hours post transfection (passage 0), which was used for further infections. Viral titers were determined by end-point dilution on HeLa cells. The 3A sequence was analyzed by Sanger sequencing on PCR products amplified from cDNA of infected cells using primers 5′-CTCACAGCCTCACTGTCTAC-3′ and 5′-TGCTCATGTCCACCGGCAAG-3′.

### CRISPR

AGO2 knockout clones were generated by transfection of HeLa cells with pCRISPR-hCAS9-1xgRNA-Puro plasmid^58^, selection under puromycin selection, and single cell cloning as described previously^30^. The online tool from the Zhang laboratory (http://crispr.mit.edu/) was used to design guide RNAs^59^. The following guides were used: for AGO2 KO1 guide 1 F 5′- ACCGCCAAAGTCGGGTCTAGGTGG-3, guide 1 R 5′-AACCCACCTAGACCCGACTTTGGC-3′ and guide 39 F 5′-ACCGACTCTCCTCGGGCACTTCTC-3′, guide 39 R 5′-AACGAGAAGTGCCCGAGGAGAGTC-3′ and for AGO2 KO2 guide 51 F 5′-ACCGACCGCGTCTGCAATGTGACC-3′, guide 51 R 5′-AACGGTCACATTGCAGACGCGGTC-3′ and guide 55 F 5′-ACCGCCCATGTTTACAAGTCGGAC-3′, guide 55 R 5′-AACGTCCGACTTGTAAACATGGGC-3′. Cells surviving puromycin selection were seeded in single-cell dilutions in 96-well plates, cultured, and expanded into flasks. Genomic DNA was isolated from individual cell clones and the presence of a deletion in the AGO2 gene was analyzed on agarose gel after a diagnostic PCR using primers flanking the expected target sites (described below). The deletions were validated by Sanger sequencing. HeLa cells transfected with the same CRISPR reagents lacking guide RNA sequences were used as controls in all experiments, except for the experiments in which RNAi suppression by 3A was analyzed (reporter assay and western blot).

### DNA sequencing

Genomic DNA of AGO2 KO clones was isolated using Zymo Research Quick-gDNA MiniPrep (D3024). The AGO2 sequence flanking the targeted region was PCR amplified using the following primer combinations: AGO2_1 F 5′-GTTTGATCACCAATGAGTTG-3′ and AGO2_1 R 5′-CTGTGTCTCAATACAAAAAC-3′ for KO1 cells, AGO2_2 F 5′-CCTATGAAAACTGATTCTCG-3′ (outside the targeted region) in combination with AGO2_2 R 5′-TGCCCGTCGGAGTGACAGTG-3′ (outside the targeted region) or AGO2_3 R 5′-CAGTGCCATTTGTATCTGAC-3′ (inside the targeted region) for KO2 cells. The same primers were used for Sanger sequencing.

### SDS PAGE and western blot

WT or AGO2 KO HeLa cells were seeded in 6-well plates at a density of 1.5 × 10^5^ cells/mL in 2 mL medium. Cells were collected in lysis buffer (40 mM Tris-HCl, 150 mM NaCl, 10 mM EDTA, and 1% NP-40), rotated at 4 °C for 30 minutes, and centrifuged for 30 minutes at 16,000 *g*. Cell lysates were resolved on a 10% SDS PAGE gel, blotted to nitrocellulose membranes, and probed with the indicated antibodies. Primary antibodies α-AGO2 (Merck 11A9) and α-actin (Merck A5441) were diluted 1:500 and 1:30,000, respectively, in PBS containing 0.1% Tween and 2.5% nonfat dry milk. Secondary antibodies IRdye α-Rabbit (LI-COR biosciences 926–32211) and IRdye α-mouse (LI-COR biosciences 926–32220) were diluted 1:5,000 in PBS containing 0.1% Tween, 2.5% nonfat dry milk, and 0.01% SDS. Expression of 3A from p3A transfected HeLa cells was analyzed using an anti-myc antibody (Thermo Scientific PA1-981) at a 1:1000 dilution.

### RT qPCR

RNA was isolated from Poly (I:C) stimulated and virus infected samples using RNA-Solv reagent (Omega R630-02). RNA was treated with DNase I and cDNA was generated using TaqMan reverse transcription reagents (Thermofisher technologies N8080234) according to the manufacturer’s instructions. Gene expression was measured by qPCR using the GoTaq qPCR master mix (Promega A6002) and the following primers: IFNβ F 5′-GCTTGGATTCCTACAAAGAAGCA-3′, IFN R 5′- ATAGATGGTCAATGCGGCGTC-3′, ISG15 F 5′-CGCAGATCACCCAGAAGATCG-3′, ISG15 R 5′-TTCGTCGCATTTGTCCACCA-3, actin F 5′-CCTTCCTGGGCATGGAGTCCTG-3′ and actin R 5′-GGAGCAATGATCTTGATCTTC-3′. To measure viral RNA, the following primers were used: SINV F 5′-ACTTTAGCAGTGGCCGTGAC-3′, SINV R 5′-TCATGCTGCAAGCAAAAATC-3′, YFV17D F 5′-ACGGATAGCGGGAAAGTTATTC-3′, YFV17D R 5′-GGATACCAACACCCATCACTAC-3′, CVB3 F 5′-CTGCCAATGGTGACATACGTGA-3′, CVB3 R 5′-ATCACAACCGACTGCACTGC-3′, EMCV F 5′-GCCGAAAGCCACGTGTGTAA-3′ and EMCV R 5′-AGATCCCAGCCAGTGGGGTA-3′. Relative gene expression was analyzed using the ΔΔC_T_ method^60^.

### RNAi reporter assay

RNAi reporter assays were performed as described earlier^30^. WT and AGO2 KO HeLa cells were seeded at a density of 1 × 10^4^ cells/well in a 96-well format and transfected with a combination of plasmids encoding luciferase reporters, shRNA expression plasmids, and, where indicated, AGO2 expression plasmids using FuGENE6 transfection reagent (Promega E2693) in DMEM without serum. The following expression plasmids were used: pcDNA6-Ago2-myc or pcDNA6-Ago2-D597A-myc (100 ng/well)^11^, pGL3-TK (firefly luciferase, 100 ng)^30^, pRL-TK (Promega, Renilla luciferase as a normalization control, 100 ng), and either 75 ng of plasmid encoding Fluc shRNAs (pCR-U6-luc2) or a control shRNA targeting an earlier generation luciferase construct (GL2), which is inactive against the GL3 luciferase sequence due to three mismatches (control; pCR-U6-luc1)^30^. 48 hours post transfection, cells were lysed using Passive Lysis Buffer, and luciferase reporter activity was quantified using the Dual-Luciferase Reporter Assay (Promega E1960).

To assess RNAi suppression by picornavirus 3A proteins, HeLa cells were seeded at a density of 7.5 × 10^4^ cells per well in a 24-well plate. The next day, cells were transfected with 500 ng of p3A(CVB3)-myc, p3A(EV-A71)-myc, p3A(PV1)-myc, pcDNA3.1Puro NoV B2, or control plasmid^50,51,61,62^ using FuGENE 6 (Promega E2691). Two days after the initial transfection, cells were transfected with 100 ng pGL3-TK, 100 ng pRL-TK, and 75 ng of either pCR-U6-luc1 (GL2) or pCR-U6-luc2 (GL3) using FuGENE 6. Cells were lysed the next day in 100 µl of Passive Lysis Buffer and luminescence was measured using the Dual-Luciferase Reporter Assay (Promega).

### Small RNA library preparation and analysis

For CVB3 libraries, HeLa and HEK293T cells were seeded at a density of 1 × 10^6^ cells in a T25 flask and infected the next day using passage 0 virus stock at an MOI of 0.01. For EMCV libraries, HeLa cells (WT and AGO2 KO2) were seeded at a density of 1.5 × 10^5^ cells per well in a 6-well plate and two wells were infected with EMCV the next day at an MOI of 1. Total RNA was isolated from CVB3 infected cells at 24 hpi and from EMCV infected cells at 8 or 10 hpi using RNA-Solv reagent (Omega Biotek R630-02). 25 µg of total RNA was separated on a 15% acrylamide/7 M urea/0.5x TBE gel, and small RNAs in the size range of 19 to 33 nt was cut from gel, eluted, precipitated in 100% ethanol, and dissolved in 11 µl nuclease-free water. 5 µl of the purified small RNAs was used as input for library preparation using the NEBNext Small RNA Library Prep (NEB E7300S/E7580S). PCR products were size purified on a 6% acrylamide/1x TBE gel and quantified using the Agilent 2100 Bioanalyzer System. Libraries were pooled and sequenced on an Illumina Hiseq 4000 machine by GenomEast Platform (Strasbourg, France). The small RNA reads were mapped to the CVB3 Nancy strain (GenBank accession: M33854.1) using Bowtie (Galaxy tool version 1.1.2)^63^, allowing 1 mismatch in the first 32 nucleotides of each read. The genome distribution of virus-mapping small RNAs (21–23 nt) was obtained by plotting the number of 5′ ends of the reads at each position of the viral genome. All reads were normalized to total library size (RPM, reads per million). For miRNA analysis, the small RNA reads were mapped without mismatches to the human genome GRCh38 assembly using Bowtie (version 1.1.2). The genome coordinates for mature miRNAs (using coordinates in hsa.gff3from miRBase v22) were intersected with the mapped reads to find all mature miRNAs in the datasets (using bedtools intersect intervals version 2.27.0; -wa). miRNAs with an average count of >50 reads in the four libraries were considered for the correlation analyses of miRNAs in WT and AGO2 KO cells.

## Supplementary information

Supplementary information

## Data Availability

Newly generated small RNA datasets have been deposited in the NCBI Sequence Read Archive repository under accession number PRJNA550031. Other datasets are included in this published article and its Supplementary Information files or available from the corresponding author on reasonable request.
