# Supplementary material for: No evidence for viral small RNA production and antiviral function of Argonaute 2 in human cells
Source: Sci Rep. 2019 Sep 24;9:13752. doi: 10.1038/s41598-019-50287-w (PMC6760161; doi:10.1038/s41598-019-50287-w)
Supplement: Supplementary file 1 — Supplementary information [file 41598_2019_50287_MOESM1_ESM.pdf]

Supplementary figures

No evidence for viral small RNA production and antiviral function of Argonaute 2 in human cells

Susan Schuster[1], Gijs J. Overheul[1], Lisa Bauer[2], Frank J.M. van Kuppeveld[2], Ronald P. van Rij[1*]

[1] Department of Medical Microbiology, Radboud University Medical Center, Radboud Institute for Molecular Life Sciences, Nijmegen, The Netherlands
[2] Department of Infectious Diseases & Immunology, Faculty of Veterinary Medicine, Utrecht University, Utrecht, The Netherlands

*Corresponding author:
Dr. Ronald P. van Rij, Department of Medical Microbiology, Radboud University Medical Center, Radboud Institute for Molecular Life Sciences, PO Box 9101, 6500 HB Nijmegen, The Netherlands, Tel.: +31-24-3617574, e-mail: Ronald.vanRij@radboudumc.nl

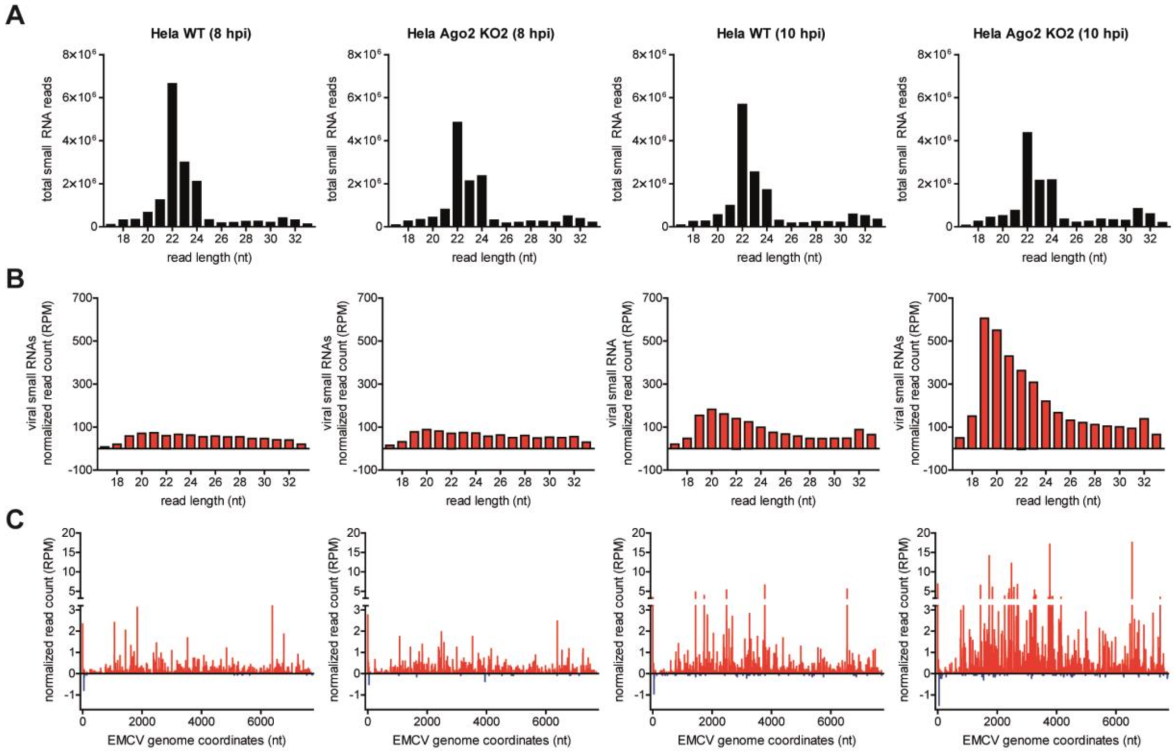

Supplementary Figure S1. Small RNA profiles in EMCV infected WT and AGO2 deficient HeLa cells. (**A**) Size profiles of total small RNAs in libraries from WT and AGO2 KO2 cells infected with EMCV (MOI 1) at 8 and 10 hours post infection (hpi), as indicated. (**B**) Size profiles of small RNAs mapping to the positive viral RNA strand (red) or negative RNA strand (blue) of EMCV. (**C**) Genome distribution of 21-23 nt small RNAs across the EMCV genome. 5' positions of normalized reads are plotted. Reads were mapped to the viral genome allowing one mismatch and normalized to the total library size.

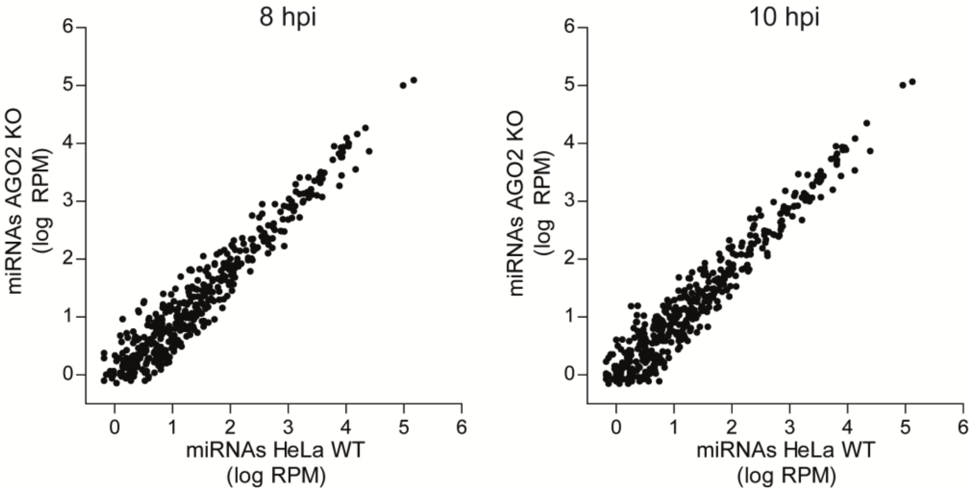

Supplementary Figure S2. miRNA levels in EMCV infected WT and AGO2 KO2 HeLa cells. Scatter plots representing miRNA levels normalized to library size of EMCV infected cells at 8 hpi (left panel) and 10 hpi (right panel). The mean of the 8 and 10 hpi time points is reported in Fig. 2B.

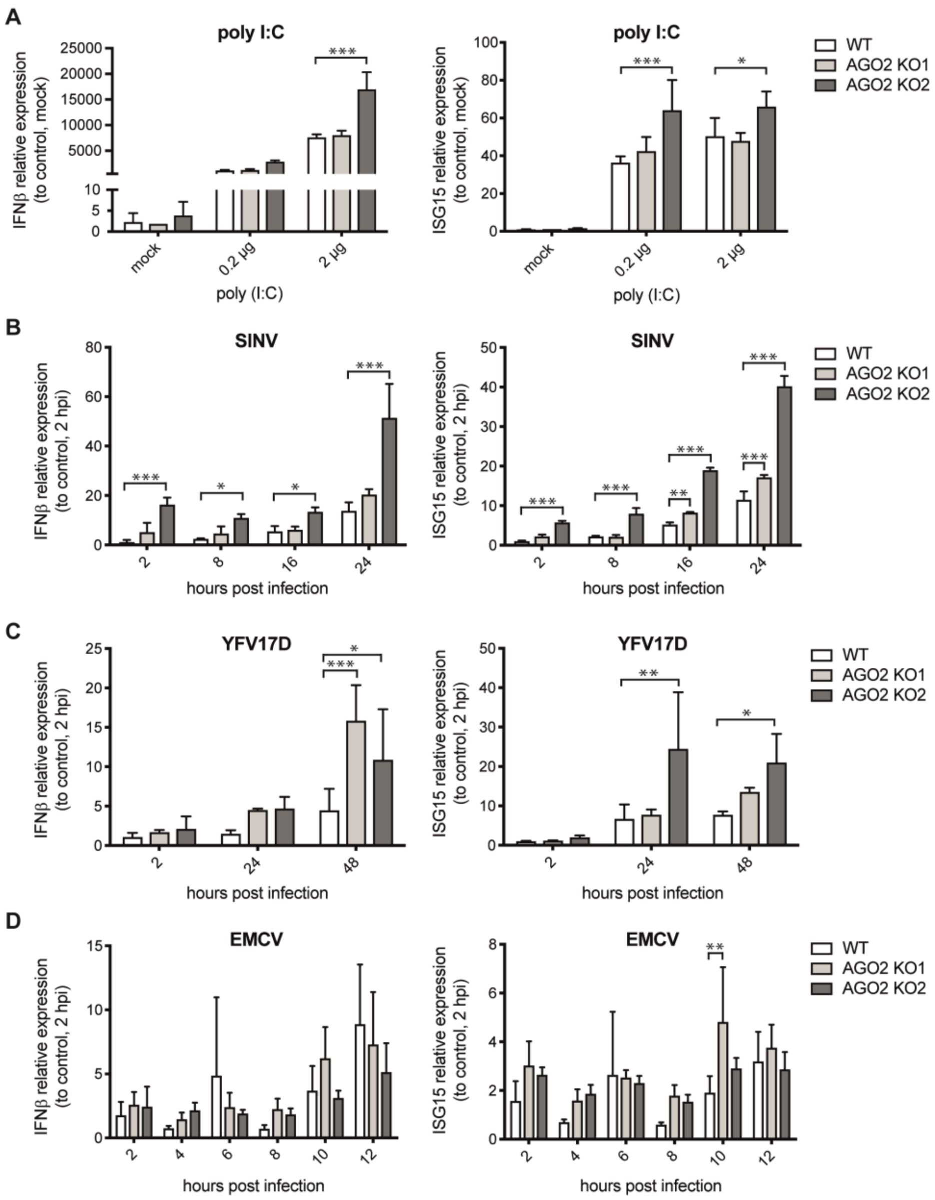

Supplementary Figure S3. Activation of innate immune signaling in the absence of Argonaute 2. Expression of IFNβ (left panels) and ISG15 (right panels) upon activation of WT (white bars) and AGO2 KO HeLa cells (two clones, light and dark gray bars) with (**A**) poly I:C, 200 ng or 2 μg per well, (**B**) Sindbis virus (SINV, MOI 0.01), (**C**) yellow fever virus (YFV17D, MOI 0.1), and (**D**) encephalomyocarditis (EMCV, MOI 1). RNA was quantified by RT-qPCR, data were normalized to the house-keeping gene actin and expressed relative to WT control cells that were mock treated (A), or to WT control cells at 2 hpi (B,C,D). Data are presented as means and SD of three biological replicates. One experiment representative of three experiments is shown. ANOVA, * $P < 0.05$, ** $P < 0.01$, *** $P < 0.001$. hpi, hours post infection.

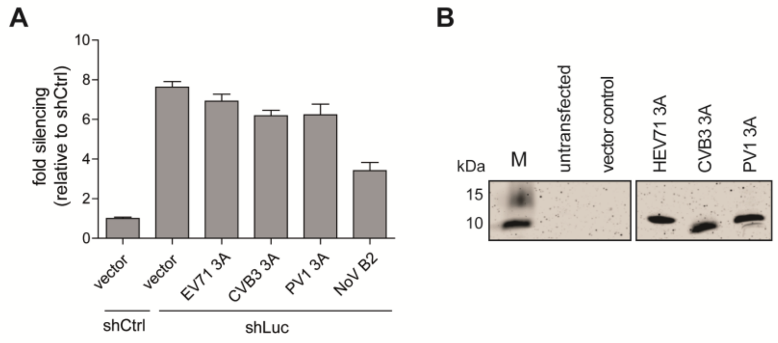

Supplementary Figure S4. RNAi suppression assay for picornavirus 3A proteins. (**A**) RNAi reporter assay for the 3A protein of human enterovirus-71 (HEV71), Coxsackievirus B3 (CVB3), poliovirus-1 (PV1), and as a positive control the Nodamura virus (NoV) B2 protein. The indicated expression plasmids were transfected into HeLa cells, followed by transfection with expression plasmids encoding firefly luciferase (Fluc) and Renilla luciferase (Rluc) and a plasmid expressing an shRNA that targets either Fluc (shGL3) or control shRNA (shCtrl). Fluc counts were normalized to Rluc and presented as fold silencing relative to control shRNA. Means and SD (n=3 replicates) are presented. (**B**) Western blot analysis of myc epitope-tagged 3A protein. The indicated myc-tagged 3A expression plasmids were transfected into HeLa cells and expression was analyzed on western blot using a myc antibody. The full-length blot is presented in Supplementary Fig. S5.

A. Figure 1C

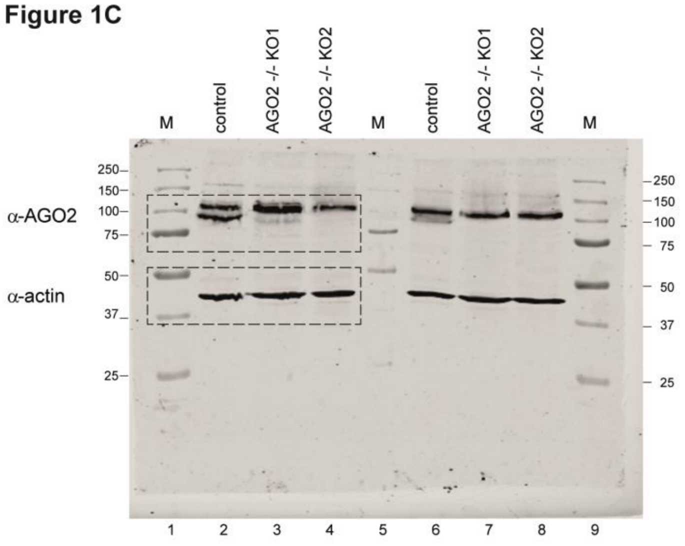

B. Figure S4B

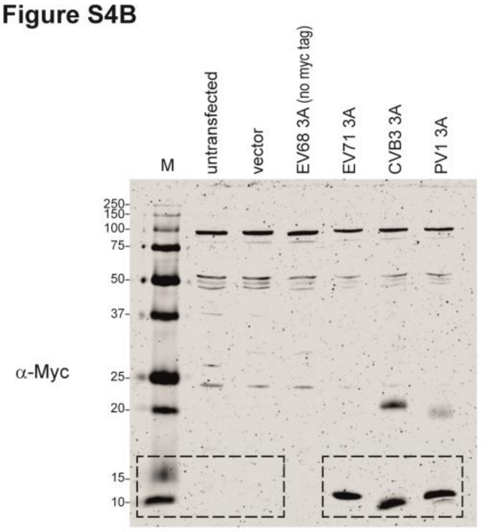

Supplementary Figure S5. Full-length gel images of western blots of (A) Figure 1C, and (B) Figure S4B. Dashed boxes indicate the regions used for the figures. (**A**) The same samples have been loaded twice following denaturing at 60°C for 15 minutes (lanes 2-4) or at 95°C for 5 minutes (lanes 6-8).
